# Detection of high-risk pregnancies in low-resource settings: a case study in Guatemala

**DOI:** 10.1186/s12978-019-0748-z

**Published:** 2019-06-11

**Authors:** Patricia Hanna Crispín Milart, Ignacio Prieto-Egido, Cesar Augusto Díaz Molina, Andrés Martínez-Fernández

**Affiliations:** 1 Obstetrics and Gynecology Unit, Fundación Alcorcón University Hospital, C/ Budapest, 1, 28922 – Alcorcon, Madrid, Spain; 2 Universidad Rey Juan Carlos, Camino del Molino s/n, Fuenlabrada, 28943 Spain; 3 Tulasalud – non-governmental organization. Alta Verapaz, Guatemala, 6ta. Calle 3-42 Zona 4, Cobán, Alta Verapaz Guatemala

**Keywords:** Prenatal care, Maternal mortality, Obstetric ultrasound, Low-income countries, Rural areas

## Abstract

**Background:**

Maternal and neonatal mortality is still very high at a global level, even though its reduction is a goal established among the Sustainable Development Goals by the United Nations. In order to improve prenatal care to address this challenge, this article proposes a strategy to detect and refer high risk pregnancies in rural setting through a portable ultrasound system combined with blood and urine strip tests.

**Methods:**

The Healthy Pregnancy project was conceived as a single, explanatory and positivist case study, with a sample of ten thousand pregnant women attended by itinerant nurses of the Departments of Alta Verapaz and San Marcos. These nurses were trained and equipped with 31 portable ultrasound, and blood and urine tests to detect common obstetric pathology. Moreover, two obstetricians were responsible for remotely supervising the quality of prenatal care. Target communities were selected by the Health Directorates of the public health system from those that had the highest maternal mortality in previous years.

**Results:**

The project attended to 10,108 women in 2 years and 3 months. 55 twin gestations (0.54%) were diagnosed. Non-cephalic presentation was found in 14.87% of the pregnant women attended from week 32 onwards. 20 patients were referred for non-evolutive gestation. An 11.08% prevalence of anemia was detected. Urine infections were diagnosed in 16.43% of the cases. Proteinuria was detected in 2.6% of patients, but only 17 of them presented high blood pressure and were therefore referred with a suspected pre-eclampsia.

**Discussion:**

The results obtained indicate that an intervention of these characteristics makes it possible to improve the quality of care of rural pregnant women in low and middle-income countries.

**Conclusion:**

The results show that with suitable equipment, training, and supervision, the nursing staff in charge of care in rural areas can identify and refer most of the obstetric risks in time, which may contribute to the reduction of maternal mortality.

**Trial registration:**

This research was not registered because it is a case study in which the assignment of the medical intervention was not at the discretion of the investigators.

**Electronic supplementary material:**

The online version of this article (10.1186/s12978-019-0748-z) contains supplementary material, which is available to authorized users.

## Plain ENGLISH summary

Maternal and neonatal mortality still represents a major problem in most low and middle-income countries, particularly in rural areas. Most of these deaths could be prevented with adequate care before, during and after childbirth. However, this is a difficult task due to the lack of specialists and resources, as well as the time required to reach the hospital in emergency cases. This article analyses a project that implements a screening protocol for the early detection of common obstetric pathologies. The protocol is based on a portable ultrasound system complemented with fast blood and urine strip tests, which, used together, facilitate the detection of the most frequent obstetric risks. Nurses who visit rural communities are trained in the use of these systems and the application of the screening protocol. In the case of high risk being detected, the nurses will refer the patient to a higher-level facility, to receive adequate care before any complications appear. This protocol has been applied to a sample of 10,108 pregnant women in rural areas of the Alta Verapaz and San Marcos Departments (Guatemala). The pregnancy risks detected in this case study are coherent with the literature review, showing that it is possible to improve prenatal care in rural settings of low and middle-income countries.

## Background

The gap in terms of access to adequate sexual and reproductive care represents a violation of human rights which affects millions of women throughout the world, particularly those living in low and middle-income countries. This situation led to the death of 303,000 women in 2015 due to pregnancy-associated causes. Of these women, 99% lived in low and middle-income countries, where the likelihood of death due to such causes is 30 times higher than in high-income countries [1]. The reduction in the global maternal mortality is a goal established by the United Nations in the Sustainable Development Goals [2] (SDGs), however, nowadays, the reduction rate is not enough in order to reach this goal [3]. The majority of maternal deaths occur in rural settings, and most of them could be prevented with adequate medical care before, during and after childbirth. However, the lack of resources and qualified staff, as well as the difficulty in referring or transferring patients, makes it difficult to ensure the right to quality care in rural communities, where only 56% of births will receive medical care by qualified staff [4].

Previous works have identified the importance of taking good-quality prenatal care to rural communities, in order to reduce maternal mortality through the training of the staff working in these areas [5]. The guide “WHO recommendations on antenatal care for a positive pregnancy experience” recommends that adequate prenatal care should include assessment of anemia, asymptomatic bacteriuria, gestational diabetes, HIV, tuberculosis, and syphilis as part of the maternal assessment [6]. For fetal assessment, an ultrasound is recommended before week 24 to identify multiple gestation, congenital anomalies, estimate gestational age, fetal malpresentation and placenta previa. It also mentions that ultrasound is the most accurate screening tool not widely available in LMIC.

In particular, adequate prenatal control should detect a prevalence of asymptomatic bacteriuria based on that which is described in the literature, between 2 and 15% [7]. Women with untreated asymptomatic bacteriuria have a higher risk of developing pyelonephritis, low weight at birth and pre-term childbirth. Multiple pregnancies are associated with pre-term birth and a 7 times greater risk of neonatal death [8]. In a recent prospective study, the multiple pregnancy rate for Guatemala was 0.8% [9]. The rate of detection of non-cephalic presentation from week 32 onwards should be between 7 and 16% [10]. Regarding placenta previa, the rate of incidence is 0.5% [11].

However, the lack of resources in rural communities makes quality care difficult, because it is not possible to conduct conventional blood tests due to the lack of laboratories and the fact that it is not feasible to maintain the cold chain. Moreover, ultrasound tests are not viable, due to the lack of electricity, equipment, and trained staff. To overcome these barriers and reduce maternal mortality, several authors have put forward innovative solutions based on m-health (mobile devices supporting the practice of medicine and public health) and telehealth (the remote use of ICTs, Information and Communication Technologies in the health sector). Through these solutions, they try to improve data collection [12, 13], reinforce the follow-up of pregnant women through SMS [14], facilitate the work of midwives by providing support in decision making [15], evaluate ultrasound tests in an asynchronous manner [16] and improve post-childbirth follow-up through SMS to prevent HIV transmission [17]. However, the articles published have only analyzed their impact on process indicators, and different reviews [18, 19] have not been able to find studies that evaluate its effects on maternal mortality. The only study found that assessed the impact of an intervention on maternal mortality [20] is focused on hospitals of reference and cannot be extrapolated to rural regions with a lack of resources.

The authors of this study have previously published a study using a portable ultrasound system complemented with dry blood tests and fast urine strips [21]. In total, 762 pregnant women were tested with the system, and there were no maternal deaths in that sample. Since these results showed a promising trend, it was decided that a second phase of the project would be conducted to multiply the number of pregnant women tested by ten. This article presents the results obtained from this second phase of the project, which we have called Healthy Pregnancy.

## Methods

The Healthy Pregnancy project was proposed as a “case study,” given the limitations posed when using resources from Multilateral (IADB) and Bilateral (AECID and USAID) Agencies of International Cooperation for Human Development. These agencies forced us to intervene in the areas with the highest maternal mortality in Guatemala and to use all funds in what, in an experimental study, would be the intervention group. We could not have a control group, which limited the research methodology and the scope of it.

Therefore, a single explanatory and positivist case study was designed, with a sample of ten thousand pregnant women attended by the itinerant nurses of the Departments of Alta Verapaz and San Marcos. This sample is not able to provide a statistical generalization, but rather, an analytical one, that will allow us to find new relationships and to induce new proposals that can be contrasted in future experimental studies.

Four research questions were defined:

1. Is the addition of portable ultrasound and blood and urine analysis to the prenatal control performed by nurses in the rural areas of Guatemala a feasible, effective and efficient intervention for the diagnosis of common obstetric pathology?
2. With an adequate training and reinforcement program, and an appropriate information system, is it possible to train nurses to perform basic obstetric ultrasound and obtain high-quality ultrasound studies in most of the care provided?
3. Is the identification of pregnant women with pathology or obstetric risk, and their early reference, an opportunity to reduce maternal mortality?
4. Do pregnant women accept the prenatal control offered? Do the health personnel accept and incorporates it into their daily work?

In order to answer these questions, thirty-one Healthy Pregnancy kits were distributed (for conducting ultrasound, blood and urine tests) among the public health system nurses who conduct prenatal monitoring in the departments (through regular visits every two or three months). The objective was the early identification of patients with obstetric risk and their non-urgent referral to health centers in order to receive appropriate treatment. In addition to the distribution of equipment, the project carried out training in the use of the diagnostic tools and patient management, as well as remote quality control by two obstetricians located in an urban area.

**Fig. 1:**
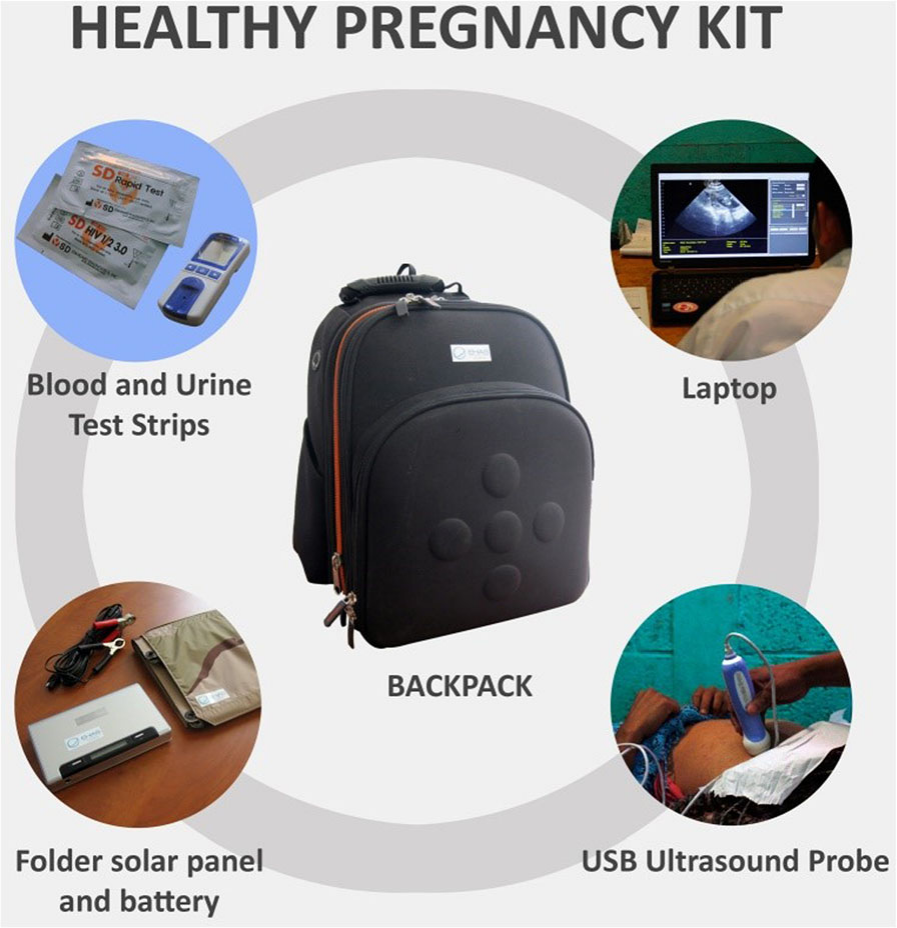
Materials of the prenatal control kit *Healthy Pregnancy.* A backpack containing a laptop, USB ultrasound probe, blood and urine rapid tests, two external batteries and a folding solar panel

The Healthy Pregnancy Kit (Fig. 1) is a backpack that contains a laptop, a USB ultrasound probe, a foldable solar panel, an external battery and materials for blood and urine tests. It includes reactive strips with immediate results for HIV, syphilis and HBV, a hemoglobin-meter, a glucometer and urine dipsticks. Each laptop features a Health Information System based on a free software platform (OpenMRS), specially adapted for this project, which allows the recording of data and imaging for each patient tested.

To implement the project 70 nurses were trained through theoretical and practical sessions to evaluate fetal number, cardiac activity, presentation, fetal biometry and placental location. Moreover, two obstetricians were available for the project to supervise the quality of prenatal care. The Health Directorates of the public health system selected target communities among those that had the highest maternal mortality in previous years.

For the evaluation, the following percentages were calculated: fetal malpresentation (defined as all non-cephalic presentations from week 32 onwards), multiple pregnancies, cases of placenta previa, the prevalence of anemia, prevalence of UTI, preeclampsia and positive cases in the serology test. Surveys among pregnant women and nurses were also conducted to evaluate the acceptability of the project.

Maternal Mortality Rate in the Project (MMRP) was defined as the ratio between women who died and had been managed by the project, and the total number of pregnant women managed, multiplied by 100,000.

Ethical approval for the research protocol was received in July 2012 by the Interagency Committee of Research and Innovation in Health of Alta Verapaz, and all patients gave their written consent to take part in the study before conducting the tests.

## Results

**Fig. 2:**
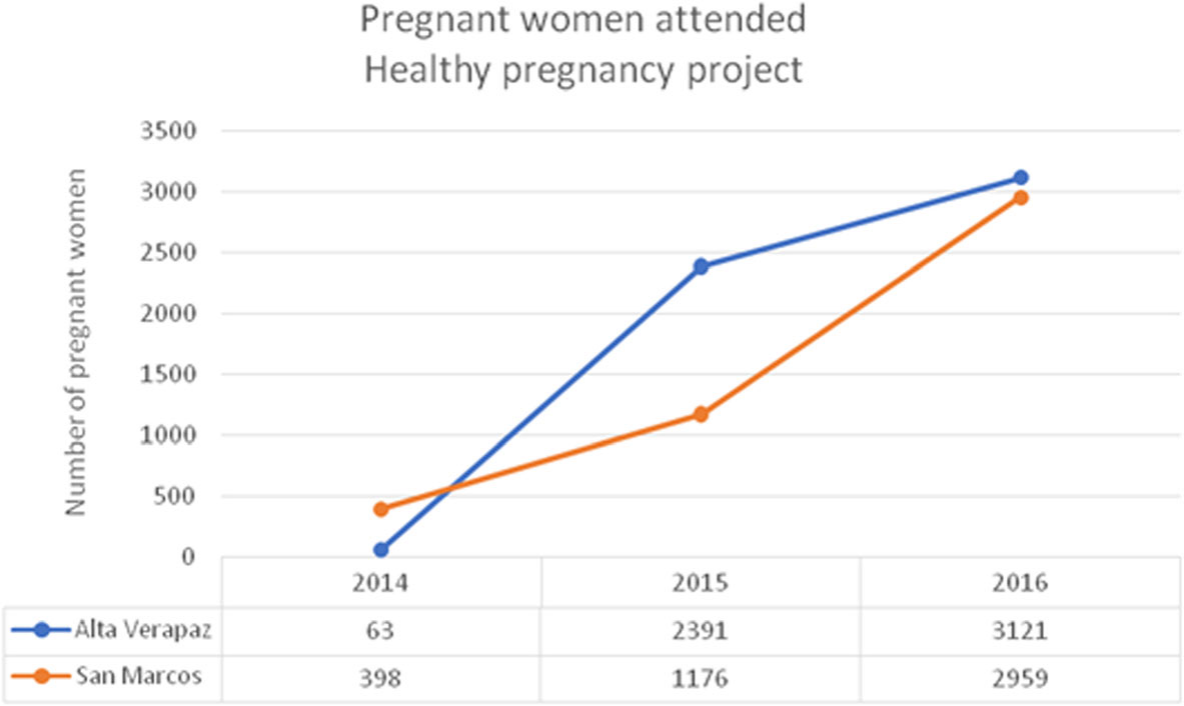
Pregnant women tested per year and department

From October 2014 to December 2016, the Healthy Pregnancy project teams tested 10,108 pregnant women. Of those, 55 revealed twin pregnancies and 39 had two pregnancies during the study. The pregnant women tested per department are detailed in Fig. 2. In both departments, we see a clear increase in patient recruitment as the project progressed.

Table 1 shows the demographic details of the population. The mean age of women examined was 25.20 years (SD 6.82), with 11 being the minimum and 47 the maximum age. In our series, adolescent pregnancy reached 21.67% in the San Marcos Department and 24.63% in Alta Verapaz. Six hundred and eighty women (6.7%) had a background of 1 or more stillbirths.

**Table 1:** Demographic Details

	Alta Verapaz Department	San Marcos Department
Total number of women tested	5596	4512
Age^a^	25.05	25.39
- <=15 years old	161 (2.88%)	98 (2.17%)
- 16–19 years old	1218 (21.76%)	880 (19.50%)
- 20–39 years old.	4058 (72.50%)	3386 (75.04%)
- > = 40 years old	159 (2.84%)	136 (3.01%)
Parity^a^		
- Nulliparous	1662 (29.70%)	1236 (27.39%)
- 1–4 previous childbirths	3147 (56.24%)	2679 (59.38%)
- 5–9 previous childbirths	731 (13.06%)	561 (12.43%)
- > = 10 previous childbirths	44 (0.79%)	30 (0.66%)
Past history of 1 or more stillbirths	364 (6.5%)	316 (7%)
Gestational age at first visit^a^		
- 1st trimester	409 (7.30%)	525 (11.63%)
- 2nd trimester	2111 (37.72%)	2187 (48.47%)
- 3rd trimester	3071 (54.87%)	1798 (39.84%)

*Information on ages was not available for 0.27% of pregnant women (*N* = 12). Childbirth data was unknown for 18 cases, and in 7 cases women had unknown amenorrhea, and the ultrasound test was not conclusive

Although the Pregnancy Monitoring Guidelines by the MSPAS recommend recruitment and monitoring of pregnant women from the first trimester, over 90% of women were seen after 14 weeks, and almost half (48%) did not have their first visit until the third trimester. The group that started the latest pregnancy control was that of parous women over 39 years old in the department of Alta Verapaz, where 65.4% had their first visit after 28 weeks. Gestational age presented in Table 1 was estimated with the sonographic study.

### Hemoglobin test

There was an evaluation of 89.96% of the total population using hemoglobin-meters (a shortage caused the low coverage). A level of hemoglobin < 11 g/dL was detected in 11.08% of the 10,108 women. Table 2 shows the details of anemia results according to severity. The anemia rate detected in the Alta Verapaz Department was 14.94 and 6.29% in the San Marcos Department. For the tested patient group, we found an association between adolescent pregnancy and the diagnosis of anemia (OR 1.29, *p* < 0.05); we did not find differences between nulliparous and parous women. Patients diagnosed with anemia were treated and cases with Hb < 8 g/dl were also advised to attend their health center for follow-up.

**Table 2:** Hemoglobin and urine test results

	Alta Verapaz (*N* = 5596)		San Marcos (*N* = 4512)		Total *N* = 10,108	
*HEMOGLOBIN TEST*						
Mild anemia (Hb < 11 g/dL)	605	10.81%	181	4.01%	786	7.78%
Moderate anemia (Hb 7–9,9 g/dL)	224	4.00%	101	2.24%	325	3.22%
Severe anemia (Hb < 7 g/dL)	7	0.13%	2	0.04%	9	0.09%
Normal Hb	3976	71.05%	3997	88.58%	7973	78.87%
Not tested	784	14.01%	231	5.12%	1015	10.04%
Total anemia cases	836	14.94%	284	6.29%	1120	11.08%
*URINE TESTS*						
Bacteriuria / UTI	1181	21.10%	487	10.79%	1668	16.50%
(+) Proteinuria + elevated BP (Pre-eclampsia)	9		8		17	0.17%
Normal results	3846	68.72%	3922	86.92%	7768	76.84%
Not tested	508	9.08%	89	1.97%	597	5.91%

### Urine tests

In total, 9512 urine tests were conducted, and urine infections were diagnosed in 1668 cases (16.43%). We considered a test to be pathological when 2 or 3+ leukocytes and/or positive nitrites were present. The results with 1+ in leukocytes were classified as sample contamination to improve test specificity. Proteinuria was detected in 2.6% of patients (263 results); of these, 17 presented high blood pressure and were therefore diagnosed as suspected pre-eclampsia. Another 180 samples tested positive for proteinuria with normal blood pressure, and since they presented findings compatible with urinary tract infection (UTI), they were dealt with accordingly. The urine test results are shown in Table 2. Patients with a diagnosis of UTI were treated, and patients with suspected pre-eclampsia were referred to the health center.

### Glucose test

A common practice for screening gestational diabetes is to conduct the O’Sullivan Test (administration of 50 g of glucose and measuring glycaemia rise after 1 h). Given the difficulty in rural settings to find fasting patients, the project used the same cut-off point as the O’Sullivan Test (140 mg/dl). The objective was to screen patients at risk and then refer them to a higher-level healthcare center for diagnosis. It was decided that the benefits of screening with this approach were higher than not conducting it all. In total, 9942 tests were conducted. In the case of Alta Verapaz, 10 (0.08%) patients were identified with glucose > = 140, and 17 (0.38%) in San Marcos.

### Screening for serologies

There were 9304 tests conducted for HBV, 8878 for syphilis and 9373 for HIV. Of these, 24 patients showed positive results and were advised to undergo a confirmation test at their health center. In total, 13 cases were confirmed: 7 for HBV, 2 for syphilis and 4 for HIV. Seven (7) tests were false positives, and at the data collection deadline, there was no confirmed answer for 4 patients.

### Other results

Fifty-five (55) twin pregnancies were detected: 38 in the Alta Verapaz Department (0.67%) and 17 in San Marcos (0.37%). A consensus was reached in the healthcare protocol to refer all non-cephalic cases > 32 weeks for delivery to healthcare centers. In total, there were 454 women: 329 in Alta Verapaz and 125 in San Marcos. The overall rate of fetal malpresentation was 4.49% of the women tested, which corresponded to 14.87% of the pregnant women attended from week 32 onwards.

Twenty (20) patients were referred for non-viable pregnancy: 2 stillbirths (25 and 26-week-old fetuses), 3 late miscarriages and 3 cases suspected to be molar pregnancies, 2 anembryonic pregnancies, 3 miscarriages during the first trimester and 7 patients with > 10-week amenorrhea where no pregnancy was detected. Seven women were diagnosed and referred because of placenta previa in the third trimester.

Even though the detection of fetal malformations was not one of the objectives of the project, nurses received basic notions of fetal morphology during their training. It was considered logical for the numerous basic studies to enable nurses to develop skills for detecting severe malformations. As a result, 11 pregnant women were referred for suspected fetal malformation: 5 suspected anencephalies, 4 findings of hydrocephaly and 2 cases of gastroschisis (one in a hydropic abortion at 17 weeks).

**Fig. 3:**
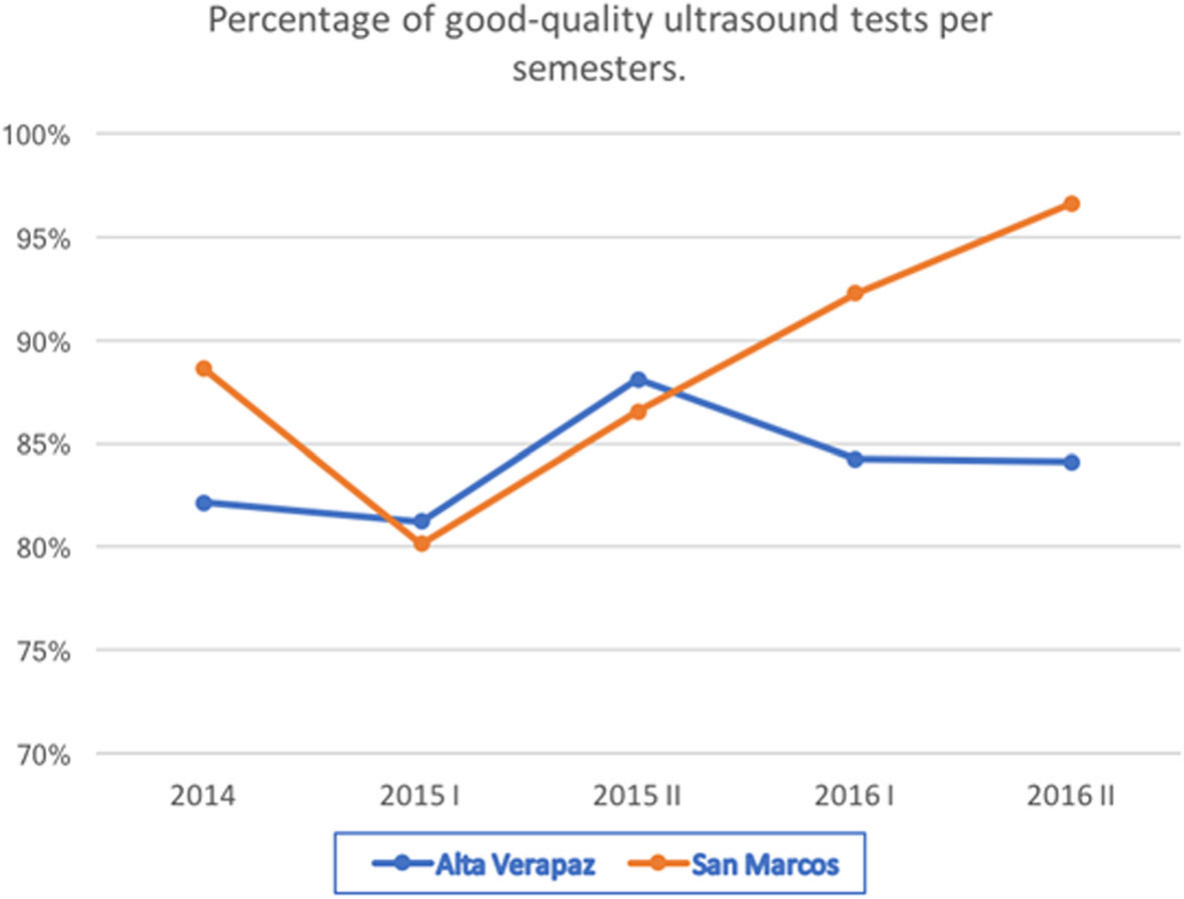
Percentage of good-quality ultrasound tests per semesters

Quality control was conducted on 100% of the 10,108 tests by two obstetricians using the information system of the project. Quality assessment for the ultrasound tests took place at 4 levels: (1) The four biometry images are correct; (2) At least 3 acceptable images; (3) Only one acceptable image, or (4) No quality ultrasound. It was considered that a good ultrasound test should receive a score of (1) or (2). Figure 3 shows the progression per semester of the proportion of good-quality ultrasound tests. A clear improvement was observed during the project for nurses in the San Marcos Department, and there was a sustained level of quality in the Alta Verapaz Department.

For the calculation of MMRP, 8995 women with an estimated date of delivery (EDD) up to December 31, 2016 were considered. Therefore, the study excluded 1113 women with a subsequent EDD (620 in SM and 493 in AV).

The total number of pregnant women who died was 9, which resulted in a MMRP of 100.05 per 100,000 live births. There were 4 deaths recorded in the SM Department in our group of 3892 women and 5 cases in the group of 5103 women in the Alta Verapaz Department.

A total of 192 pregnant women were interviewed to evaluate the acceptability of the Healthy Pregnancy Kit - 96 in each of the two departments. 50 of the 192 women surveyed were nulliparous, and among the remaining women (142) only 49 (34.5%) had access to an ultrasound in their previous pregnancies. To the question “what rating would you give in general to the attention received?” a total of 98% of the women interviewed answered with the following results: good (54%), very good (25%) or excellent (19%), therefore it can be said that pregnant women are satisfied with the care received. It is important to highlight that 99.4% of women answered that they would recommend the tests to other women in their communities.

Regarding the nurses that participated in the project, they agree (25%) or strongly agree (75%) that the equipment provided by the project is easy to use. In addition, they also agree (31%) or strongly agree (69%) that the follow-up and advice received from the specialist who performs quality control have improved their training and helped them to better define the diagnosis. Overall, 100% of the nurses are satisfied with the project and believe that it contributes to their professional growth and that it also helps to improve maternal health care of their areas.

The complete Healthy Pregnancy kit has a cost of $ 5000, and it makes it possible to carry out 300 prenatal controls per year. Estimating a repayment period of 4 years, this is $ 4.1 per woman attended. The cost of attending each woman during a complete gestation amounts to $ 28.7 per pregnant woman since we need to add to the previous value: strip tests ($ 8.7), personnel and repairs.

## Discussion

Alta Verapaz and San Marcos are two of the 3 departments in Guatemala with the highest maternal mortality. The Healthy Pregnancy project started working in the districts with the highest number of deaths of pregnant women and newborn. After 3 years, this study has verified a MMRP of 97.98 in Alta Verapaz and 102.77 in San Marcos.

Without the objective of making a statistically significant comparison, Table 3 shows the mortality data in our project, compared to the official MMR data in 2015 (the latest official figure) for both departments, published in the Report of Activities by the MSPAS [22]. In Table 3, we also show the MMR data for the country of Guatemala, obtained from the same source.

**Table 3:** Maternal Mortality

	Alta Verapaz		San Marcos		Guatemala
	MSPAS	Healthy Pregnancy	MSPAS	Healthy Pregnancy	MSPAS
Maternal Mortality (Pregnant women tested)	47	5 (5103)	31	4 (3892)	354
Live births	28.312		22.441		319.358
MMR	166.01	97.98	138.14	102.77	110.86

The results of this work confirm the trend already detected in the first pilot project carried out in 2012 and 2013 [21].

It is essential to highlight that case studies, such as the one carried out in this research, cannot be used to contrast known theories or proposals, but are instead useful for proposing new research hypotheses that will have to be tested in future experimental studies. The intention, therefore is not to achieve a statistical generalization, but, rather, an analytical one, generalizable only to other cases that present theoretical conditions similar to the present. Even so, the facts described in this paper hint at the possibilities offered by these types of projects for reducing maternal mortality among pregnant women in rural areas of low and middle-income countries.

It was particularly relevant for this study to confirm if the technology, training and new procedures implemented by the Healthy Pregnancy project allow rural nurses to detect most of the obstetric complications. This project has identified a rate of 16.43% of asymptomatic bacteriuria which is slightly higher than the one usually reported (between 2 and 15%). The rate of multiple pregnancies identified was 0.54%, which is marginally lower than the one registered for Guatemala (0.8%). The rate of detection of fetal malpresentation from week 32 onwards (14.87%) was consistent with that previously described in the background section, which is between 7 and 16%. Regarding the glucose tests, the results were far lower than the ones described in the literature, and therefore a modification in the project is being conducted to carry out the test only on fasting women (if they have eaten previously, the test is not performed).

The “Healthy Pregnancy” project enables detection of some important obstetric complications in time, facilitating the referral of pregnant women at a high risk of losing their own lives or the lives of their newborn children. In this sense, we consider that this project contributes to the improvement of prenatal control offered to women in rural areas.

## Conclusion

Rural areas of Guatemala still show high levels of maternal mortality and the public health system lacks the resources to offer ultrasounds, blood tests and specialized medical attention to all women living in these areas. The Healthy Pregnancy initiative proposes a strategy to improve prenatal care in this context by providing equipment, training, and supervision to nurses who are already responsible for prenatal controls in such areas. This case study shows that, with this strategy, the nursing staff can identify and refer most of the obstetric risks in time, which may contribute to the reduction of maternal mortality.

The EHAS Foundation is currently working to extend the project to other Guatemala departments, as well as to other countries in Latin America and Africa. Nowadays, the project has been wholly transferred to the Health Directorates of the Alta Verapaz and San Marcos departments, and patient care is conducted practically without any external financial support, under the responsibility of the relevant Public Health Management Departments.

Additional file 1 contains a Spanish version of this original article.

## Additional file

Additional file 1: Translation of this article into Spanish. (DOCX 376 kb)

## Data Availability

The datasets used and/or analyzed during the study are available from the corresponding author upon reasonable request.

## Abbreviations

BP: Blood Pressure
EDD: Estimated Date of Delivery
Hb: Hemoglobin
HBV: Hepatitis B Virus
HIV: Human Immunodeficiency Virus
ICT: Information and Communication Technologies
MMRP: Maternal Mortality Rate Project
MSPAS: Ministry of Public Health and Social Assistance
SDG: Sustainable Development Goals
UTI: Urinary Tract Infections
