# Supplementary material for: Detection of high-risk pregnancies in low-resource settings: a case study in Guatemala
Source: Reprod Health. 2019 Jun 11;16:80. doi: 10.1186/s12978-019-0748-z (PMC6560738; doi:10.1186/s12978-019-0748-z)
Supplement: Supplementary file 1 — Translation of this article into Spanish. (DOCX 376 kb) [file 12978_2019_748_MOESM1_ESM.docx]

**RESEARCH**

**TÍTULO.**

Detección de embarazos de alto riesgo en entornos de escasos recursos: estudio de caso en Guatemala.

**AUTORES.**

1. Patricia Hanna Crispín Milart: Unidad de Ginecología y Obstetricia, *Hospital Universitario Fundación Alcorcón.* Dirección: C/ Budapest, 1, 28922 – Alcorcón, Madrid-España. Email: phcrispin@fhalcorcon.es
2. Ignacio Prieto-Egido (Corresponding Author): Profesor ayudante doctor en la *Universidad Rey Juan Carlos.* Dirección: Departamental-III, D-201, Camino del Molino s/n, 28943 - Fuenlabrada (Madrid) - España. Email: ignacio.prieto@urjc.es
3. Cesar Augusto Díaz Molina: Asociación Tulasalud – ONG. Alta Verapaz, Guatemala. Dirección: 6ta. Calle 3-42 Zona 4, Cobán Alta Verapaz, Guatemala. Email: cesaraugusto@tulasalud.org
4. Andrés Martínez Fernández: Profesor titular en la *Universidad Rey Juan Carlos.* Dirección: Departamental-III, D-213, Camino del Molino s/n, 28943 - Fuenlabrada (Madrid) - España. Email: andres.martinez@urjc.es

**Corresponding author**: Ignacio Prieto-Egido.

**RESUMEN**

**INTRODUCCIÓN**

La mortalidad materna y neonatal sigue mostrando niveles muy elevados a nivel mundial, aunque su reducción es un objetivo establecido por las Naciones Unidas entre los Objetivos de Desarrollo Sostenible. Con el fin de mejorar la atención prenatal y enfrentar este desafío, este artículo propone una estrategia para detectar y referir los embarazos de alto riesgo en entornos rurales a través de un sistema de ecografía portátil combinado con pruebas rápidas de sangre y orina.

**METODOLOGÍA**

El proyecto Embarazo Saludable fue concebido como un estudio de caso único, explicativo y positivista, con una muestra de diez mil mujeres embarazadas atendidas por enfermeras itinerantes de los departamentos de Alta Verapaz y San Marcos. Estas enfermeras fueron entrenadas y equipadas con 31 ecógrafos portátiles y con pruebas rápidas de sangre y orina para detectar patologías obstétricas comunes. Además, dos médicos obstetras supervisaron de forma remota la calidad de la atención prenatal realizada. Las comunidades de intervención fueron seleccionadas por las Direcciones de Salud del sistema público de salud entre aquellas que tuvieron la mortalidad materna más alta en años anteriores.

**RESULTADOS**

El proyecto atendió a 10.108 mujeres en 2 años y 3 meses. Se diagnosticaron 55 gestaciones gemelares (0,54%). Se detectó presentación no cefálica en el 14,87% de las mujeres embarazadas atendidas desde la semana 32 en adelante. 20 pacientes fueron referidas por gestación no evolutiva. Se encontró una prevalencia de anemia del 11,08%. Se diagnosticó infección de orina en un 16,43% de los casos. Se detectó proteinuria en el 2,6% de las pacientes, ~~pero solo~~ 17 de ellas presentaron hipertensión arterial y, por lo tanto, fueron referidas con sospecha de preeclampsia.

**DISCUSIÓN**

Los resultados obtenidos indican que con una intervención de estas características es posible mejorar la calidad de la atención rural de las mujeres embarazadas en países de ingresos bajos y medios.

**CONCLUSIÓN**

Los resultados muestran que con el equipo, la capacitación y la supervisión adecuados, el personal de enfermería a cargo de las áreas rurales puede identificar y referir a tiempo la mayoría de los riesgos obstétricos, lo que puede contribuir a la reducción de la mortalidad materna.

**Registro del ensayo**: esta investigación no se registró porque es un estudio de caso en el que la asignación de la intervención médica no fue a discreción de los investigadores.

**Palabras clave:** Atención prenatal, mortalidad materna, ecografía obstétrica, países de bajos ingresos, zonas rurales.

**RESUMEN BÁSICO**

La mortalidad materna y neonatal sigue siendo un grave problema en la mayor parte de los países de ingresos bajos y medios, particularmente en las zonas rurales. La mayoría de estas muertes se podrían prevenir con una atención adecuada antes, durante y después del parto. Sin embargo, esta es una tarea difícil debido a la falta de especialistas y de recursos, así como ~~el~~ al elevado tiempo requerido para llegar al hospital en casos de emergencia. Este artículo analiza un proyecto que emplea un protocolo de cribado para la detección temprana de patologías obstétricas comunes. El protocolo se basa en un sistema de ecografía portátil complementado con pruebas rápidas de sangre y orina que, en conjunto, facilitan la detección de los riesgos obstétricos más frecuentes. Las enfermeras que visitan las comunidades rurales están formadas para el uso de estos sistemas y aplicar el protocolo de cribado. Cuando se detecte un caso de alto riesgo, las enfermeras referirán a la paciente a un centro de mayor nivel para que reciba la atención adecuada antes de que aparezcan complicaciones. Este protocolo se ha aplicado a una muestra de 10.108 mujeres embarazadas en áreas rurales de los departamentos de Alta Verapaz y San Marcos (Guatemala). Los riesgos de embarazo detectados en este estudio de caso son coherentes con la literatura, lo que demuestra que es posible mejorar la atención prenatal en entornos rurales de países de ingresos bajos y medios.

**Introducción**

La brecha de acceso a una adecuada atención sexual y reproductiva representa una violación de los derechos humanos que afecta a millones de mujeres en todo el mundo, en particular a las que viven en países de ingresos bajos y medios. Esta situación provocó en el año 2015 la muerte de 303.000 mujeres debido a causas asociadas con el embarazo. De estas mujeres, el 99% vivía en países de ingresos bajos y medios, donde la probabilidad de morir por tales causas es 30 veces mayor que en los países de ingresos altos [1]. La reducción de la mortalidad materna mundial es un objetivo establecido por las Naciones Unidas en los Objetivos de Desarrollo Sostenible (ODS) [2], sin embargo, en la actualidad la tasa de reducción no es suficiente para alcanzar este objetivo [3]. La mayoría de las muertes maternas ocurren en entornos rurales, y gran parte de ellas podrían prevenirse con una atención médica adecuada antes, durante y después del parto. Sin embargo, la falta de recursos y de personal cualificado, así como la dificultad para referir o transferir pacientes, hace que sea difícil garantizar el derecho a una atención de calidad en las comunidades rurales, donde solo el 56% de los partos recibirá atención médica por parte de personal cualificado [4].

Trabajos anteriores han identificado la importancia de llevar atención prenatal de buena calidad a las comunidades rurales, a fin de reducir la mortalidad materna a través de la capacitación del personal que trabaja en estas áreas [5]. La guía "Recomendaciones de la OMS sobre atención prenatal para una experiencia positiva del embarazo" recomienda que una atención prenatal adecuada debe incluir en la evaluación materna: valoración de anemia, bacteriuria asintomática, diabetes gestacional, VIH, tuberculosis y sífilis [6]. Para la evaluación fetal se recomienda una ecografía antes de la semana 24 para identificar la gestación múltiple, estimar edad gestacional, identificar anomalías congénitas, malpresentación fetal y placenta previa. También menciona que la ecografía es la herramienta de detección más precisa, aunque no se encuentra ampliamente disponible en países de ingresos bajos y medios.

En particular, un adecuado control prenatal debe detectar una prevalencia de bacteriuria asintomática cercana a lo descrito en la literatura, entre el 2% y el 15% [7]. Las mujeres con bacteriuria asintomática no tratada tienen un mayor riesgo de pielonefritis, bajo peso al nacer y parto prematuro. Los embarazos múltiples están asociados con el parto prematuro y un riesgo 7 veces mayor de muerte neonatal [8]. En un reciente estudio prospectivo, la tasa de embarazo múltiple en Guatemala fue de 0,8% [9]. La tasa de detección de presentación no cefálica a partir de la semana 32 en adelante debe estar entre el 7% y el 16% [10]. En cuanto a la placenta previa, la tasa de incidencia es del 0,5% [11].

Sin embargo, la falta de recursos en las comunidades rurales dificulta la calidad de la atención, porque no es posible realizar análisis de sangre convencionales debido a la falta de laboratorios y a que no es posible mantener la cadena de frío. Además, las pruebas de ecografía no son realizables debido a la falta de electricidad, equipamiento y personal capacitado. Para superar estas barreras y reducir la mortalidad materna, varios autores han propuesto soluciones innovadoras basadas en mhealth (dispositivos móviles que apoyan la práctica de la medicina y la salud pública) y telemedicina (el uso remoto de las TIC, las Tecnologías de la Información y la Comunicación en el sector de la salud). A través de estas soluciones intentan mejorar la recopilación de datos [12] [13], refuerzan el seguimiento de las mujeres embarazadas a través de SMS [14], facilitan el trabajo de las matronas brindando apoyo en la toma de decisiones [15], evalúan pruebas de ecografía de forma asíncrona [16] y mejoran el seguimiento posterior al parto mediante SMS para prevenir la transmisión del VIH [17]. Sin embargo, los artículos publicados solo han analizado su impacto en los indicadores de proceso, y diferentes revisiones [18, 19] no han podido encontrar investigaciones que evalúen sus efectos sobre la mortalidad materna. El único estudio encontrado que evaluó el impacto de una intervención sobre la mortalidad materna [20] se centra en los hospitales de referencia y no se puede extrapolar a las regiones rurales sin recursos.

Los autores de este artículo han publicado previamente un estudio que utiliza un sistema de ecografía portátil complementado con análisis de sangre seca y tiras de orina [21]. En total, 762 mujeres embarazadas fueron atendidas con ese sistema y no hubo muertes maternas en esa muestra. Dado que los resultados mostraron una tendencia prometedora, se decidió realizar una segunda fase del proyecto para multiplicar por diez el número de gestantes atendidas. Este artículo presenta los resultados obtenidos en esta segunda fase del proyecto, que hemos denominado Embarazo Saludable.

**METODOLOGÍA**

El proyecto Embarazo Saludable se diseñó como un estudio de caso, dadas las limitaciones planteadas al utilizar recursos de las Agencias Multilaterales (BID) y Bilaterales (AECID y USAID) de Cooperación Internacional para el Desarrollo Humano. Estas agencias priorizaron intervenir en las áreas con la mayor mortalidad materna en Guatemala y utilizar todos los fondos en lo que, en un estudio experimental, sería el grupo de intervención. No pudimos tener un grupo de control, lo que limita la metodología de investigación y el alcance de la misma.

Por lo tanto, se diseñó un estudio de caso único, explicativo y positivista, con una muestra de diez mil mujeres gestantes atendidas por enfermeras itinerantes de los departamentos de Alta Verapaz y San Marcos. Esta muestra no puede proporcionar una generalización estadística, sino analítica, que nos permitirá encontrar nuevas relaciones e inducir nuevas propuestas que puedan ser contrastadas en futuros estudios experimentales.

Se definieron cuatro preguntas de investigación:

1) ¿La adición de ecografía portátil y análisis de sangre y orina al control prenatal que realizan las enfermeras en las zonas rurales de los departamentos de Alta Verapaz y San Marcos en Guatemala es una intervención factible, eficaz y eficiente para el diagnóstico de patología obstétrica frecuente?

2) Mediante un adecuado programa de formación y refuerzo, y un sistema permanente de control de calidad, ¿es posible capacitar a enfermeras/os para realizar ecografía obstétrica básica, consiguiendo estudios ecográficos de buena calidad en la mayoría de las atenciones realizadas?

3) ¿El empleo de un adecuado Sistema de Información (diseñado especialmente para este proyecto) permite un buen control de calidad remoto de las atenciones realizadas y una correcta coordinación entre el personal rural que realiza la atención y los médicos especialistas?

4) ¿La identificación de las gestantes con patología o riesgo obstétrico, y su referencia temprana, es una oportunidad para disminuir la mortalidad materna?

5) ¿El control prenatal ofrecido es aceptado por las mujeres atendidas? ¿Lo acepta el personal de salud y lo incorpora a su trabajo diario?

Para responder a estas preguntas, se distribuyeron 31 kits de Embarazo Saludable (para realizar pruebas de ecografía, sangre y orina) a las enfermeras del sistema público de salud que realizan el seguimiento prenatal en los dos departamentos (a través de visitas periódicas cada dos o tres meses). El objetivo fue la identificación temprana de las pacientes con riesgo obstétrico y su traslado no urgente al centro de salud para recibir el tratamiento adecuado. Además de la distribución de los equipos, el proyecto formó al personal de enfermería en el uso de estas herramientas de diagnóstico y en el manejo de las pacientes, y realizó un control de calidad remoto por parte de dos médicos obstetras ubicados en áreas urbanas.

El kit de Embarazo Saludable (Figura 1) es una mochila que contiene una computadora portátil, una sonda de ecografía USB, un panel solar plegable, una batería externa, y tiras para análisis de sangre y orina. Incluye tiras reactivas con resultados inmediatos para VIH, sífilis y VHB, un hemoglobinómetro, un glucómetro y tiras reactivas para orina. Cada computadora portátil cuenta con un Sistema de Información en Salud basado en una plataforma de software libre (OpenMRS) especialmente adaptada para este proyecto, que permite el registro de datos e imágenes para cada paciente atendida.

Para implementar el proyecto, se capacitó a 70 enfermeras a través de sesiones teóricas y prácticas para evaluar: número de fetos, actividad cardíaca, presentación fetal, biometría fetal y localización placentaria. Además, dos médicos obstetras estaban disponibles para supervisar la calidad de la atención prenatal. Las Direcciones de Salud del sistema público de salud seleccionaron las comunidades objetivo entre aquellas que tuvieron la mortalidad materna más alta en años anteriores.

Para la evaluación se calcularon los siguientes porcentajes: malpresentación fetal (definida como todos los casos con presentación no cefálica a partir de la semana 32), embarazos múltiples, casos de placenta previa, prevalencia de anemia, prevalencia de ITU, preeclampsia y casos positivos en la serología. También se realizaron encuestas entre las mujeres embarazadas y el personal de enfermería para evaluar la aceptabilidad del proyecto.

La razón de mortalidad materna en el proyecto (RMMP) se definió como la división entre el número de mujeres fallecidas que fueron atendidas por el proyecto y el número total de mujeres embarazadas atendidas, multiplicado por 100.000.

La aprobación ética para el protocolo de investigación fue recibida en julio de 2012 por el Comité Interinstitucional de Investigación e Innovación en Salud de Alta Verapaz, y todas las pacientes dieron su consentimiento por escrito para participar en la investigación antes de realizar las pruebas.

**RESULTADOS**

Desde octubre de 2014 hasta diciembre de 2016, los equipos de trabajo del proyecto Embarazo Saludable evaluaron a 10.108 mujeres embarazadas. De ellas, 55 presentaron gestaciones gemelares y 39 tuvieron dos embarazos durante la investigación. Las mujeres embarazadas evaluadas en cada departamento se detallan en la Figura 2. En ambos departamentos se ve un claro aumento en el reclutamiento de pacientes a medida que el proyecto avanzaba.

La Tabla 1 muestra los detalles demográficos de la población. La edad media de las mujeres atendidas fue de 25,20 años (DS 6,82), siendo 11 la edad mínima y 47 la edad máxima. En nuestra serie, el embarazo adolescente alcanzó el 21,67% en el Departamento de San Marcos y el 24,63% en Alta Verapaz. Seiscientas ochenta mujeres (6,7%) tenían antecedentes de una o más muertes fetales.

Tabla 1. Detalles demográficos de la población.

Aunque las Guías de Atención del Embarazo del MSPAS recomiendan la captación y evaluación de las mujeres embarazadas desde el primer trimestre, más del 90% de las mujeres fueron atendidas después de la semana 14, y casi la mitad (48%) no tuvo su primera visita hasta el tercer trimestre. El grupo que inició más tarde el control del embarazo fue el de las mujeres mayores de 39 años en el departamento de Alta Verapaz, donde el 65,4% tuvo su primera visita después de la semana 28. La edad gestacional presentada en la Tabla 1 es aquella que se estimó con el estudio ecográfico.

Prueba de hemoglobina: se evaluó al 89,96% de la población total con hemoglobinómetros (la escasez de tiras causó la baja cobertura). Se detectó un nivel de hemoglobina menor de 11 g / dL en el 11,08% de las 10.108 mujeres. La Tabla 2 muestra el desglose de los resultados de anemia según gravedad. La prevalencia de anemia detectada en el departamento de Alta Verapaz fue del 14,94%, y del 6,29% en el departamento de San Marcos. Para el grupo de pacientes evaluado, encontramos asociación entre el embarazo adolescente y el diagnóstico de anemia (OR 1,29, p <0,05); No encontramos diferencias entre mujeres primíparas y el resto. A las pacientes diagnosticadas de anemia se les dio tratamiento y en los casos de Hb < 8 g / dl también se les recomendó asistir a su centro de salud para seguimiento.

Tabla 2. Resultados de las pruebas de hemoglobina y orina.

Pruebas de orina: se realizaron 9.512 exámenes rápidos de orina y se diagnosticó infección de orina en 1.668 casos (16,43%). Se consideró una prueba patológica cuando la prueba fue positiva para nitritos o positiva para leucocitos con 2 o 3+. Los resultados con 1+ en leucocitos se consideraron como contaminación de la muestra para mejorar la especificidad de la prueba. Se detectó proteinuria en el 2,6% de las pacientes (263 resultados); de estas, 17 presentaron presión arterial alta y, por lo tanto, se diagnosticaron como sospecha de preeclampsia. Otras 180 muestras dieron positivo para proteinuria con presión arterial normal y hallazgos compatibles con infección del tracto urinario (ITU), por lo que se manejaron como tal. Los resultados de los análisis de orina se muestran en la Tabla 2. Las pacientes con un diagnóstico de ITU fueron tratadas y las pacientes con sospecha de preeclampsia fueron referidas al centro de salud.

Prueba de glucosa: La prueba de O’Sullivan (administración de 50 g de glucosa y medición del aumento de la glucemia después de 1 hora) es una práctica común para cribado de diabetes gestacional. Dada la dificultad para encontrar pacientes en ayunas en el entorno rural, el proyecto utilizó el mismo punto de corte que la prueba de O’Sullivan (140 mg / dl). El objetivo era identificar a las pacientes en riesgo y luego referirlas a un centro de atención médica de mayor nivel para su diagnóstico. Se decidió que los beneficios del cribado con este enfoque eran mayores que no realizarlo. En total, se realizaron 9.942 pruebas. En el departamento de Alta Verapaz se detectaron 10 pacientes (0,08%) con glucosa por encima de los 140 mg/dL y 17 pacientes (0,38%) en el departamento de San Marcos.

Serologías: se realizaron 9.304 pruebas para VHB, 8.878 para sífilis y 9.373 para VIH. De estas, 24 pacientes presentaron resultados positivos y se les recomendó someterse a una prueba de confirmación en su centro de salud. En total se confirmaron 13 casos: 7 para VHB, 2 para sífilis y 4 para VIH. Siete pruebas fueron falsos positivos, y en la fecha límite de recolección de datos no hubo respuesta confirmada para 4 pacientes.

Se detectaron cincuenta y cinco (55) embarazos gemelares: 38 en el Departamento de Alta Verapaz (0,67%) y 17 en San Marcos (0,37%). Se acordó en el protocolo de atención referir todos los casos con presentación no cefálica a partir de las 32 semanas para que el parto tuviera lugar en centros de salud. En total se contabilizaron 454 mujeres: 329 en Alta Verapaz y 125 en San Marcos. La tasa global de malpresentación fetal fue del 4,49% de las mujeres analizadas, que corresponde al 14,87% de las embarazadas atendidas desde la semana 32 en adelante.

Se derivaron 20 pacientes por embarazo no viable: 2 muertes fetales (fetos de 25 y 26 semanas), 3 abortos tardíos y 3 casos con sospecha de embarazo molar, 2 casos con sospecha de gestación anembrionada, 3 abortos de primer trimestre y 7 pacientes con amenorrea mayor de 10 semanas a las que no se identificó gestación. Siete mujeres fueron diagnosticadas y derivadas por placenta previa en el tercer trimestre.

Pese a que la detección de malformaciones fetales no era uno de los objetivos del proyecto, el personal de enfermería recibió nociones básicas de estudio morfológico durante su entrenamiento. Parece lógico que el elevado número de estudios realizados permitieran a las enfermeras desarrollar habilidades para detectar malformaciones graves. Como resultado, se derivaron 11 mujeres por sospecha de malformación fetal: 5 sospechas de anencefalia, 4 casos de hidrocefalia y 2 casos de gastrosquisis (uno en un feto hidrópico de 17 semanas).

El 100% de los 10.108 estudios realizados pasó un control de calidad por uno de los dos médicos obstetras mediante el Sistema de Información del proyecto. Se realizó una valoración de la calidad de la ecografía en 4 niveles: (1) Las 4 imágenes de biometría son correctas; (2) al menos 3 imágenes son aceptables (3) Sólo una imagen es aceptable; o (4) ninguna de las imágenes es de buena calidad. Se consideró un estudio ecográfico de buena calidad aquellos que recibieron valoración (1) o (2). La Figura 3 presenta la progresión por semestres del porcentaje de ecografías con estudios de calidad. Se observa una clara mejoría durante el transcurso del proyecto para el personal de enfermería del departamento de San Marcos y un nivel de calidad mantenido en el departamento de Alta Verapaz.

Para el cálculo de la RMMP, se incluyeron a las 8.995 mujeres con fecha probable de parto (FPP) hasta diciembre del 2016 inclusive. Se excluyeron 1.113 mujeres (620 en SM y 493 en AV) con FPP posterior.

El total de gestantes fallecidas fue de 9, lo que resulta en una RMMP de 100,05 por 100.000 nacidos vivos. Se registraron 4 muertes en el Departamento de San Marcos en nuestro grupo de 3.892 mujeres y 5 casos en el grupo de 5.103 mujeres en el Departamento de Alta Verapaz.

Se encuestaron a un total de 192 gestantes para evaluar la aceptabilidad del sistema Embarazo Saludable, 96 en cada uno de los dos departamentos. 50 de las 192 mujeres encuestadas era nulíparas; de las 142 mujeres restantes sólo 49 (34,5%) había tenido acceso a una evaluación mediante ecografía en sus embarazos previos. A la pregunta ¿qué calificación le daría en general a la atención recibida? el 98% de las mujeres encuestadas contestó que buena (54%), muy buena (25%) o excelente (19%), lo que permite afirmar que las gestantes están satisfechas con la atención recibida. Resulta significativo resaltar que el 99,4% contestó que recomendaría las pruebas realizadas a otras mujeres en sus comunidades.

Respecto a las enfermeras que participaron en el proyecto, respondieron estar de acuerdo (25%) o muy de acuerdo (75%) en que el equipo proporcionado por el proyecto es de fácil manejo. Además, también respondieron estar de acuerdo (39%) o muy de acuerdo (61%) en que el seguimiento y la asesoría del especialista que realiza el control de calidad han mejorado su formación y les han ayudado a precisar mejor el diagnóstico. El 100% de las enfermeras y enfermeros respondieron sentirse satisfechos con el proyecto y creen que contribuye a su crecimiento profesional y consideran que ayuda a mejorar la salud materna en sus áreas.

El equipo completo de Embarazo Saludable tiene un costo de $ 5.000 y permite realizar 300 controles prenatales por año. Estimando un período de pago de 4 años, esto son $ 4,1 por mujer atendida. El costo de atención de cada embarazada durante toda su gestación asciende a $ 28,7 por mujer embarazada, ya que debemos agregar al valor anterior: tiras de sangre y orina ($ 8,7), personal y reparaciones.

**DISCUSIÓN**

Alta Verapaz y San Marcos son dos de los 3 departamentos de Guatemala con mayor mortalidad materna. El proyecto Embarazo Saludable comenzó trabajando en los distritos con las tasas más altas de mortalidad materna y neonatal. Después de 3 años, este estudio ha verificado una RMMP de 97,98 en Alta Verapaz y de 102,77 en San Marcos.

Tabla 3. Mortalidad materna.

Sin pretender realizar una comparación estadísticamente significativa, la Tabla 3 muestra los datos de mortalidad materna de nuestro proyecto, en comparación con los datos oficiales de RMM (Razón de Mortalidad Materna) en el año 2015 (la última cifra oficial) para ambos departamentos, publicados en el Informe de Actividades del MSPAS [22]. En la Tabla 3, también mostramos los datos de RMM para Guatemala país, obtenidos de la misma fuente.

Los resultados de este trabajo confirman la tendencia ya detectada en el primer proyecto piloto realizado entre 2012 y 2013.

Es importante resaltar que los estudios de caso, como el llevado a cabo en esta investigación, no pueden ser utilizados para contrastar teorías o propuestas conocidas, sino precisamente para plantear nuevas hipótesis de investigación que deberán ser contrastadas en posteriores estudios experimentales. No se pretende por tanto alcanzar una generalización estadística, sino más bien analítica, generalizable solo a otros casos que presenten condiciones teóricas similares a las del presente. Aun así, los hechos descritos en este trabajo dejan entrever las posibilidades que ofrecen estos tipos de proyectos para reducir la mortalidad materna entre mujeres embarazadas de zonas rurales en países de ingresos bajos y medios.

Fue especialmente relevante para el estudio verificar si la tecnología, el entrenamiento y los nuevos procedimientos implementados por el proyecto Embarazo Saludable permiten a las enfermeras rurales detectar la mayoría de las complicaciones obstétricas. El proyecto ha detectado una prevalencia de 16,43% de bacteriuria asintomática, ligeramente más alta que la que se reporta (entre 2% y 15%). La tasa de embarazos múltiples identificada fue de 0,54%, que es ligeramente más baja que la reportada en Guatemala (0,8%). La tasa de detección de presentación no cefálica a partir de la semana 32 (14,87%) fue consistente con la comentada previamente en la introducción, que está entre un 7% y 16%. En cuanto a las pruebas de glucosa, los resultados fueron mucho más bajos que los descritos en la literatura por lo que se está llevando a cabo una modificación en el proyecto para realizar dichas pruebas sólo a las mujeres en ayunas (si han comido anteriormente, la prueba no se realiza).

El proyecto Embarazo saludable permite la detección a tiempo de algunas complicaciones obstétricas importantes, facilitando la referencia de mujeres embarazadas en alto riesgo de perder la vida o las de sus recién nacidos. En este sentido, consideramos que este proyecto contribuye a la mejora del control prenatal de las mujeres en áreas rurales.

**CONCLUSIÓN**

Las áreas rurales de Guatemala muestran aún altos niveles de mortalidad materna y el sistema público de salud carece de los recursos para ofrecer ecografías, análisis de sangre y atención médica especializada a todas las mujeres que viven en estas áreas. La iniciativa Embarazo Saludable propone una estrategia para mejorar la atención prenatal en este contexto al proporcionar equipo, capacitación y supervisión a las enfermeras actualmente responsables de los controles prenatales en dichas áreas. Este estudio de caso muestra que, con esta estrategia, las enfermeras pueden identificar y referir la mayoría de los riesgos obstétricos a tiempo, lo que puede contribuir a la reducción de la mortalidad materna.

La Fundación EHAS está actualmente trabajando para extender el proyecto a otros departamentos de Guatemala, así como a otros países de América Latina y África. Hoy en día, el proyecto ha sido transferido completamente a las Direcciones de Salud de los departamentos de Alta Verapaz y San Marcos, y la atención de gestantes se lleva a cabo prácticamente sin ningún apoyo financiero externo, bajo la responsabilidad de los Departamentos de Salud Pública pertinentes.

**DECLARACIONES**

#### Lista de abreviaciones

PA - Presión Arterial

FPP - Fecha Probable de Parto

Hb - Hemoglobina

VHB - Virus Hepatitis B

VIH - Virus de Inmunodeficiencia Humana

TIC - Tecnología en Información y Comunicación.

RMMP - Razón de Mortalidad Materna del Proyecto

MSPAS - Ministerio de Salud Púbica y Asistencia Social

ODS - Objetivos del Desarrollo Sostenible

ITU - Infección del Trato Urinario

DS – Desviación Estándar

**Aprobación ética y consentimiento para participar.**

El protocolo de investigación fue aprobado por el Comité Interinstitucional de Investigación e Innovación en Salud del Departamento de Alta Verapaz en julio de 2012. Todas las pacientes en el grupo de intervención que fueron atendidas por enfermeras dieron su consentimiento por escrito para participar en el estudio antes de realizar las pruebas. Esta información está disponible para los editores si es requerida.

**Consentimiento para publicación.**

No aplica: no se ha publicado información individual.

**Disponibilidad de datos y materiales.**

Los datos utilizados y / o analizados durante el estudio están disponibles a través del autor correspondiente a petición razonable.

**Conflicto de interés.**

Los autores declaran que no existe ningún conflicto de interés.

**Financiación**

Este proyecto ha sido financiado por la Agencia Española de Cooperación Internacional para el Desarrollo (13-PR1-0233), el Fondo Multilateral de Inversiones del Grupo del Banco Interamericano de Desarrollo (ATM / ME-14532-GU), la Agencia de los Estados Unidos para el Desarrollo Internacional - USAID (AID-OAA-F-14-00014) y por la Universidad Politécnica de Madrid.

**Contribución de los autores**

PC diseñó las herramientas de recopilación de datos, y contribuyó a la recolección, filtrado y análisis de estos, y a la redacción y revisión del primer borrador. IP contribuyó al diseño de las herramientas de recopilación de datos, el monitoreo de la recopilación de datos, el análisis de los mismos, y la redacción y revisión del borrador del documento. CD diseñó las herramientas de recolección de datos, brindó acceso a información de salud de la Dirección de Salud de Alta Verapaz y San Marcos y monitorizó la recolección de datos. AM concibió la idea del estudio, contribuyó a la recopilación y análisis de los datos, y a la redacción y revisión del documento. Todos los autores leyeron y aprobaron el manuscrito final.

**Agradecimientos**

Agradecemos el apoyo de las Direcciones de Salud de los departamentos de Alta Verapaz y San Marcos. El estudio no hubiera sido posible sin la colaboración de los Representantes de la Dirección de Salud y el personal de enfermería de los establecimientos públicos de salud. El trabajo realizado por el personal de la Asociación TulaSalud y la Fundación EHAS también ha sido crucial. Finalmente, también nos gustaría agradecer a Javier Arturo Entrecanales y Sandrine Woitrin por su apoyo financiero para transferir la iniciativa Embarazo Saludable al sistema público para que sea sostenible a largo plazo.

**REFERENCIAS**

1. Trends in maternal mortality: 1990 to 2015: estimates by WHO, UNICEF, UNFPA, World Bank Group and the United Nations Population Division. Geneva: World Health Organization. 2015. ISBN 978 92 4 156514 1.
2. United Nations. Sustainable Development Goals (SDG). 2015. https://sustainabledevelopment.un.org/?menu=1300. Accessed 15 Jul 2018.
3. United Nations. The Millennium Development Goals Report 2015. New York 2015. ISBN 978-92-1-101320-7.
4. Lassi ZS, Das JK, Salam RA, Bhutta ZA. Evidence from community level inputs to improve quality of care for maternal and newborn health: interventions and findings. Reproductive health. 2014;11(2):S2.
5. Lassi ZS, Mansoor T, Salam RA, Das JK, Bhutta ZA. Essential pre-pregnancy and pregnancy interventions for improved maternal, newborn and child health. Reproductive health. 2014:11(1):S2.
6. World Health Organization. WHO recommendations on antenatal care for a positive pregnancy experience. World Health Organization, 2016.
7. Rogozinska E, Formina S, Zamora J, Mignini L, Khan KS. Accuracy of onsite tests to detect asymptomatic bacteriuria in pregnancy: a systematic review and meta-analysis. Obstetrics & Gynecology. 2016;128(3):495-503.
8. American College of Obstetricians and Gynecologists. Multifetal gestations: twin, triplets, and higher-order multifetal pregnancies. 2016. Practice bulletin no. 169. Obstet Gynecol, 128, e131-e146.
9. Marete I, Tenge C, Pasha O, et al. Perinatal Outcomes of Multiple Gestation Pregnancies in Kenya, Zambia, Pakistan, India, Guatemala and Argentina: A Global Network Study. American journal of perinatology. 2014;31(2):125–132. doi:10.1055/s-0033-1338173.
10. Hickok, Durlin E., et al. The frequency of breech presentation by gestational age at birth: a large population-based study. American Journal of Obstetrics & Gynecology 166.3 (1992): 851-852.
11. Silver, Robert M. Abnormal placentation: placenta previa, vasa previa, and placenta accreta. Obstetrics & Gynecology 126.3 (2015): 654-668.
12. Medhanyie AA, Little A, Yebyo H, et al. Health workers’ experiences, barriers, preferences and motivating factors in using mHealth forms in Ethiopia. Hum Resour Health 2015;3:2.
13. Van Heerden A, Norris S, Tollman S, et al. Collecting maternal health information from HIV-positive pregnant women using mobile phone-assisted face-to-face interviews in Southern Africa. J Med Internet Res 2013;15:116.
14. Ngabo F, Nguimfack J, Nwaigwe F, et al. Designing and Implementing an Innovative SMS-based alert system (RapidSMS-MCH) to monitor pregnancy and reduce maternal and child deaths in Rwanda. Pan Afr Med J 2012;13:31.
15. Vélez O, Okyere PB, Kanter AS, et al. A usability study of a mobile health application for rural Ghanaian midwives. J Midwifery Womens Health 2014;59:184-91.
16. Solano M, Kim E, Christiansen M, et al. Asynchronous telemedicine with ultrasound: Improving maternal health in developing countries. Ultrasonics Symposium (IUS), 2009 IEEE International. 2009;2316-2319.
17. Odeny TA, Bukusi EA, Cohen CR, Yuhas K, Camlin CS, McClelland RS. Texting improves testing: a randomized trial of two-way SMS to increase postpartum prevention of mother-to-child transmission retention and infant HIV testing. AIDS (London, England). 2014;28(15):2307-2312. doi:10.1097/QAD.0000000000000409.
18. Tamrat T, Kachnowski S. Special delivery: An analysis of mhealth in maternal and newborn health programs and their outcomes around the world. Matern. Child Health J. 2012;16(5):1092–1101.
19. Ag Ahmed MA, Gagnon MP, Hamelin-Brabant L, Mbemba GI, Alami H. A mixed methods systematic review of success factors of mhealth and telehealth for maternal health in Sub-Saharan Africa. mHealth. 2017;3(1) 22–22.
20. Dumont A, Fournier P, Abrahamowicz M, Haddad S, Fraser WD. Quality of care, risk management, and technology in obstetrics to reduce hospital-based maternal mortality in Senegal and Mali (QUARITE): a cluster-randomised trial. The Lancet. 2013;382:146-157.
21. Crispín PH, Diaz CA, Prieto-Egido I, Martínez-Fernández A. Use of a portable system with ultrasound and blood tests to improve prenatal controls in rural Guatemala. Reproductive health. 2016;13:110.
22. Ministry of Public Health and Social Assistance. National Center of Epidemiology. Department of Epidemiological Surveillance. Memory of Vital Statistics and Epidemiological Surveillance. Basic Indicators of Analysis of Health Situation Republic of Guatemala, 2.015. Annual Activities 2015.

**Figuras / ilustraciones Leyendas**

**Figura 1**: Componentes del de control prenatal Embarazo Saludable. Una mochila que contiene una computadora portátil, una sonda de ecografía USB, pruebas rápidas de sangre y orina, dos baterías externas y un panel solar plegable.

**Figura 2**: Mujeres embarazadas atendidas por año y departamento.

**Figura 3**: Porcentaje de pruebas de ecografía de buena calidad por semestre.

**Tablas**

**Tabla 1**. Datos demográficos.

|  | Departamento de  Alta Verapaz | Departamento de San Marcos |
| --- | --- | --- |
| Número total de mujeres atendidas | 5.596 | 4.512 |
| Edad*   - <=15 años. - 16-19 años. - 20-3años. - >= 40 años. | 25,05  161 (2,88%)  1.218 (21,76%)  4.058 (72,50%)  159 (2,84%) | 25,39  98 (2,17%)  880 (19,50%)  3.386 (75,04%)  136 (3,01%) |
| Paridad*   - Nulíparas - 1-4 partos previos. - 5-9 partos previos. - >=10 partos previos. | 1.662 (29,70%)  3.147 (56,24%)  731 (13,06%)  44 (0,79%) | 1.236 (27,39%)  2.679 (59,38%)  561 (12,43%)  30 (0,66%) |
| Antecedente de 1 o más RN muerto previo | 364 (6,5%) | 316 (7%) |
| Edad Gestacional en la primera visita   - 1er trimestre - 2do trimestre - 3er trimestre | 409 (7,30%)  2.111 (37,72%)  3.071 (54,87%) | 525 (11,63%)  2.187 (48,47%)  1.798 (39,84%) |

*El dato edad no estuvo disponible en el 0,27% de las gestantes (N = 12). El dato paridad fue desconocido en 18 casos, y en 7 casos de mujeres con amenorrea desconocida la ecografía no fue concluyente.

**Tabla 2**. Resultados de hemoglobina y pruebas de orina.

|  | Alta Verapaz  (N=5.596) | | San Marcos  (N=4.512) | | Total  N=10.108 | |
| --- | --- | --- | --- | --- | --- | --- |
| *Hemoglobina* | | | | | | |
| Anemia leve (Hb < 11 g/dL) | 605 | 10,81% | 181 | 4,01% | 786 | 7,78% |
| Anemia moderada (Hb 7- 9,9 g/dL) | 224 | 4,00% | 101 | 2,24% | 325 | 3,22% |
| Anemia severa (Hb < 7 g/dL) | 7 | 0,13% | 2 | 0,04% | 9 | 0,09% |
| Hb normal | 3.976 | 71,05% | 3997 | 88,58% | 7.973 | 78,87% |
| Prueba no realizada | 784 | 14,01% | 231 | 5,12% | 1.015 | 10,04% |
| Total de casos de anemia | 836 | 14,94% | 284 | 6,29% | 1.120 | 11,08% |
| *Prueba de orina* | | | | | | |
| Bacteriuria / ITU | 1.181 | 21,10% | 487 | 10,79% | 1.668 | 16,50% |
| (+) Proteinuria + PA>140/90 (Preeclampsia) | 9 |  | 8 |  | 17 | 0,17% |
| Resultados normales | 3.846 | 68,72% | 3922 | 86,92% | 7.768 | 76,84% |
| Prueba no realizada | 508 | 9,08% | 89 | 1,97% | 597 | 5,91% |

**Tabla 3.** Mortalidad maternal.

|  | Alta Verapaz | | San Marcos | | Guatemala |
| --- | --- | --- | --- | --- | --- |
|  | MSPAS | Embarazo Saludable | MSPAS | Embarazo Saludable | MSPAS |
| Mortalidad Materna  (Gestantes atendidas) | 47 | 5  (5.103) | 31 | 4  (3.892) | 354 |
| Nacidos vivos | 28.312 |  | 22.441 |  | 319.358 |
| **RMM** | **166,01** | **97,98** | **138,14** | **102,77** | **110,86** |


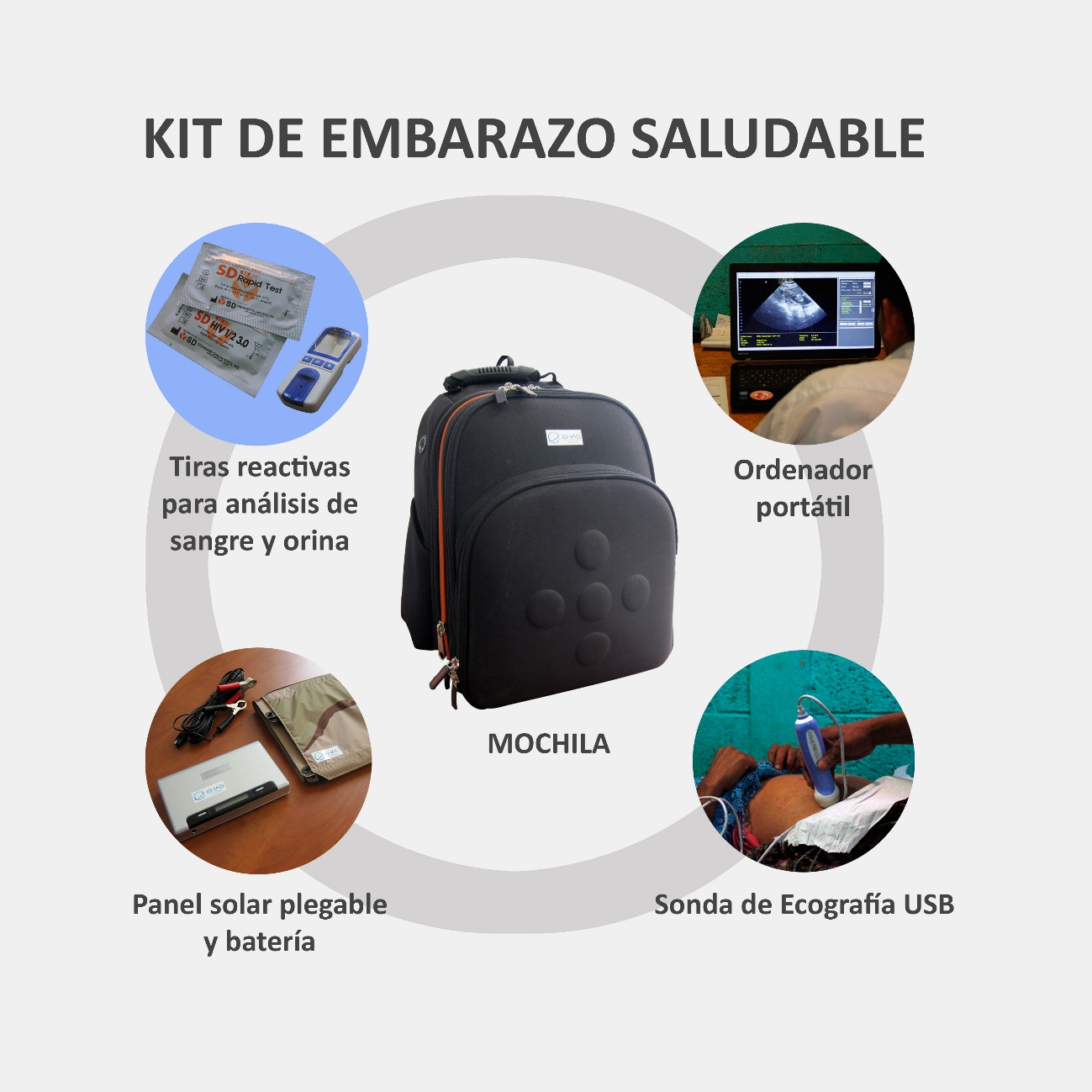


**Figura 1**

**
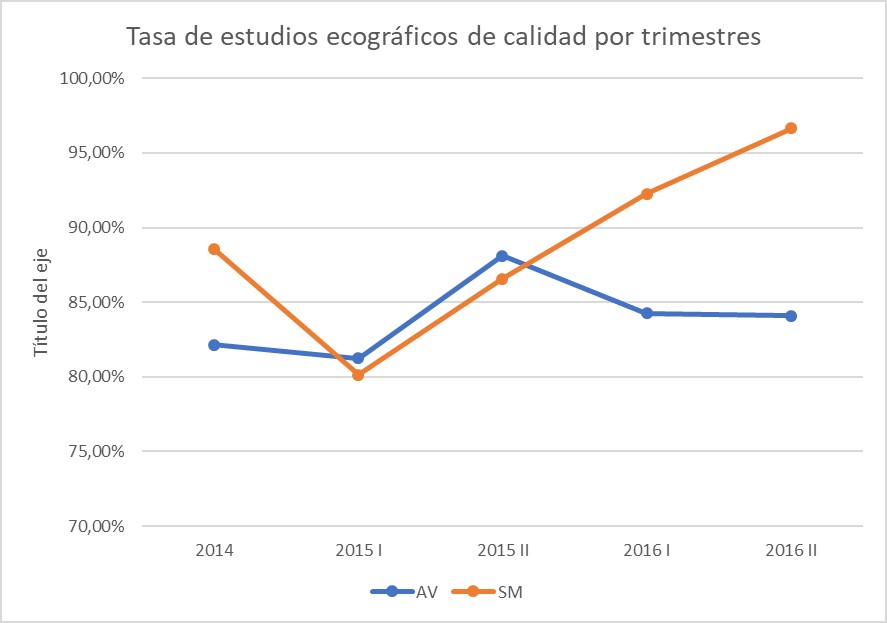
**

**Figura 2**

**
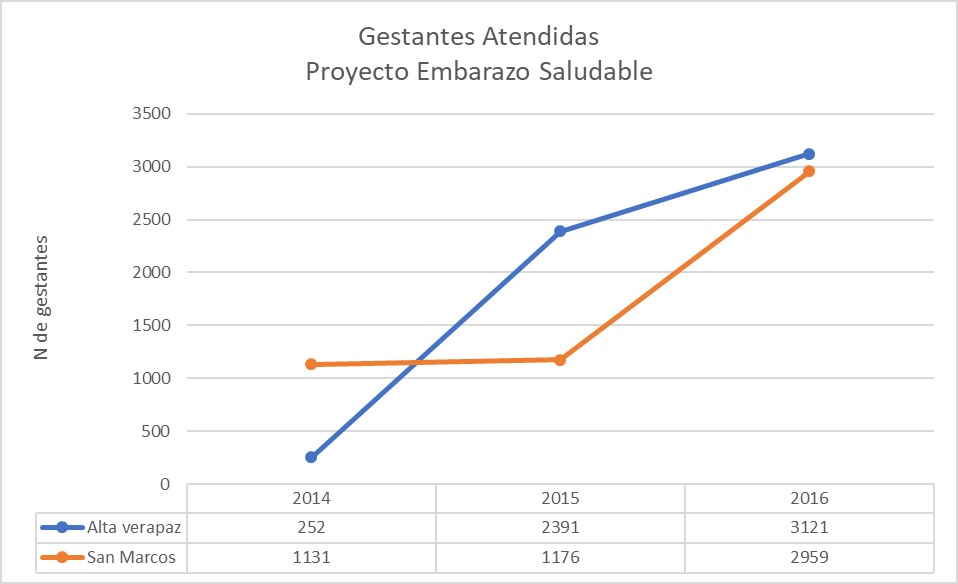
**

**Figura 3**
